# Supplementary material for: GIGANTEA recruits the UBP12 and UBP13 deubiquitylases to regulate accumulation of the ZTL photoreceptor complex
Source: Nat Commun. 2019 Aug 21;10:3750. doi: 10.1038/s41467-019-11769-7 (PMC6704089; doi:10.1038/s41467-019-11769-7)
Supplement: Supplementary file 1 — Supplementary Information [file 41467_2019_11769_MOESM1_ESM.pdf]

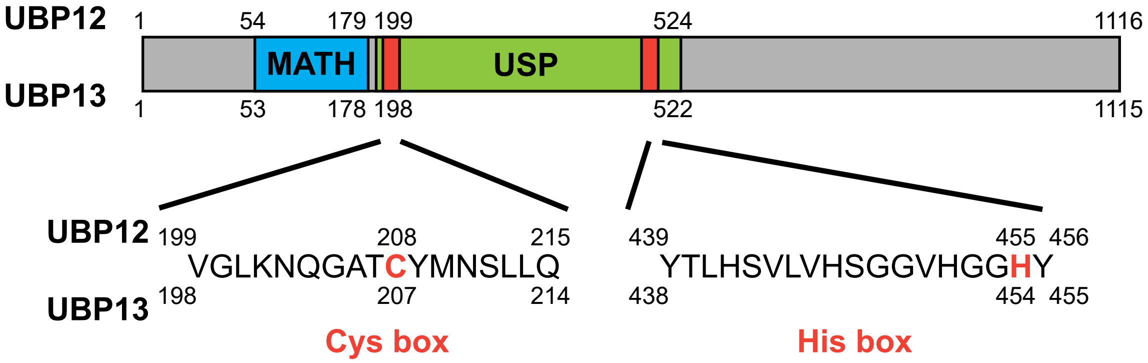

Supplementary Figure 1. Protein domain structures of UBP12 and UBP13. UBP12 and UBP13 homologous proteins contain a conserved Meprin And TRAF Homology (MATH) domain (blue) and a Ubiquitin-Specific Protease (USP) domain (green) in the N-terminus. The USP has a conserved cysteine protease enzymatic core (red): Cysteine Box (Cys box) and Histidine Box (His box). Mutations of the conserved cysteine residue to serine in the Cys box have been shown to disrupt the deubiquitylating activities of UBP12 and UBP13 24. The numbers represent the position of the amino acid sequences.

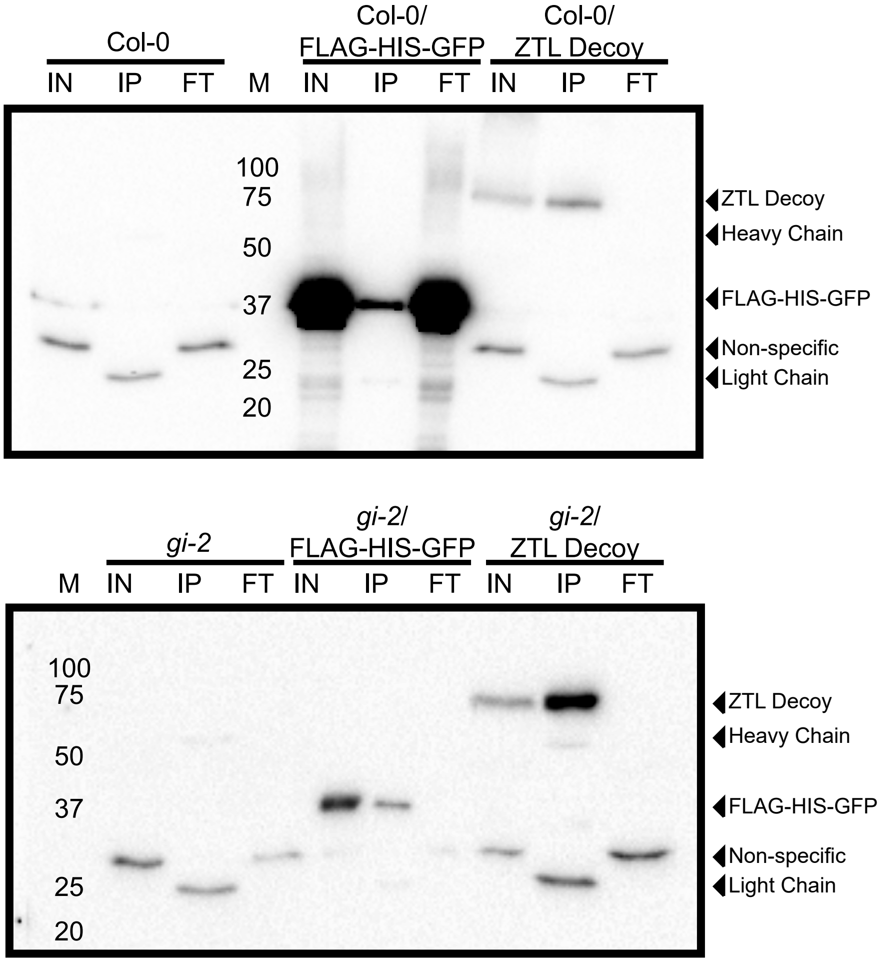

Supplementary Figure 2. Immunoprecipitation of FLAG-His-ZTL decoy in the Col-0 or *gi-2* genotypes. Immunoblots detected by anti-FLAG antibody showed that the FLAG-His-ZTL decoy and FLAG-His-GFP in the Col-0 (top panel) or *gi-2* (bottom panel) can be immunoprecipitated (IP) from the total protein extract (IN). The Col-0 (top) and *gi-2* (bottom) parental lines were negative controls. FT: flow-through.

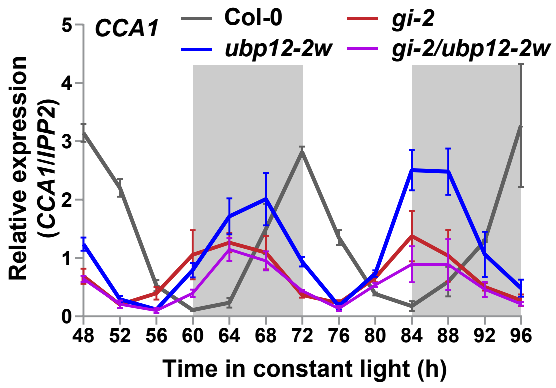

Supplementary Figure 3. Circadian expression of *CCA1* in the *gi-2/ubp12-2w* double mutant. Col-0, *ubp12-2w*, *gi-2*, and *gi-2/ubp12-2w* were entrained under 12h light/ 12h dark for 10d and then transferred to continuous light for 48h before harvest. The expression of *CCA1* was measured using qRT-PCR. The data represents the mean of the relative expression from three biological replicates with the error bars showing standard deviation. The source data are provided as a Source Data file.

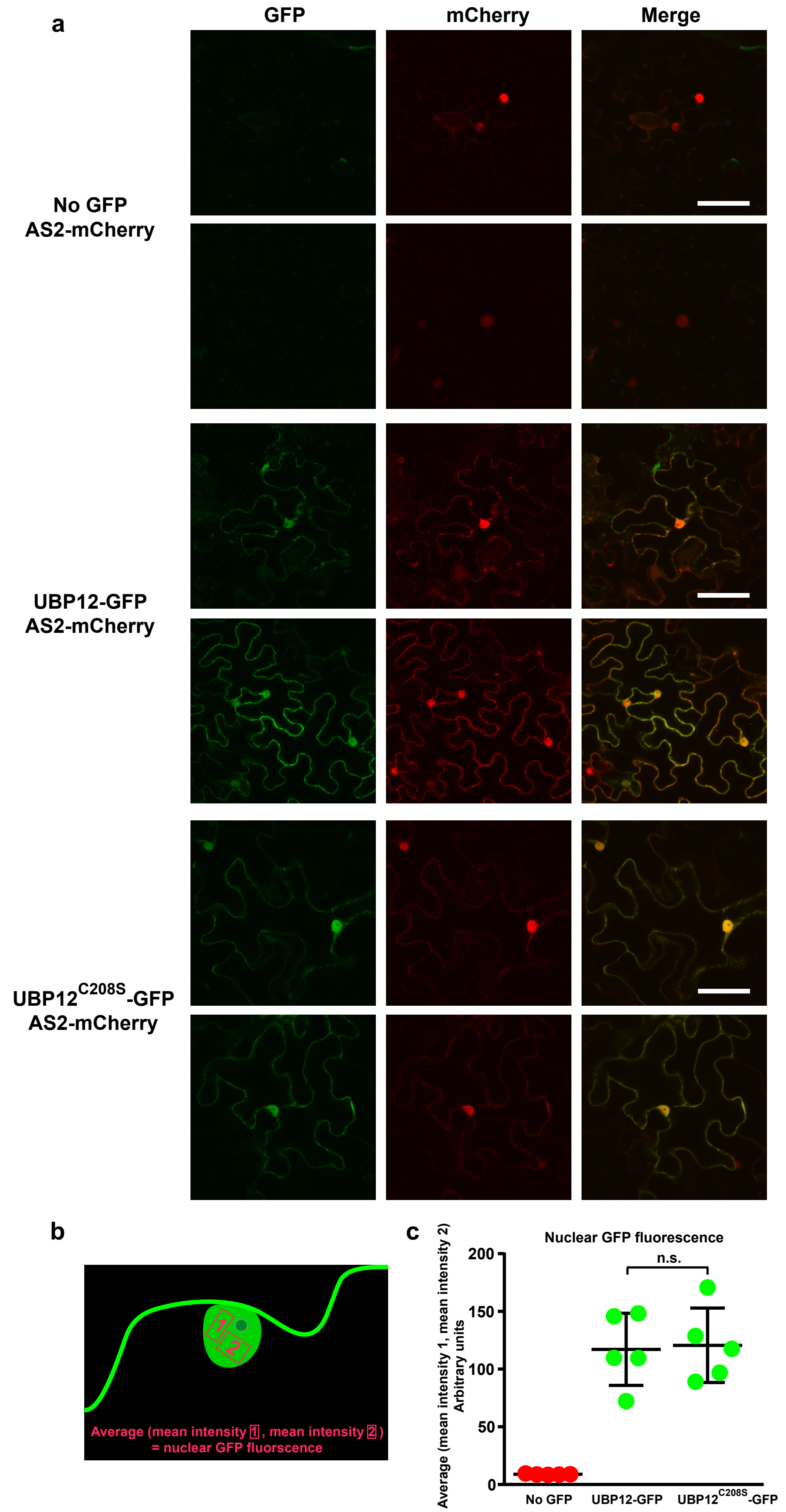

Supplementary Figure 4. The UBP12^{C208S} mutation does not alter accumulation or subcellular localization of UBP12 transiently expressed in Nicotiana benthamiana leaves. Leaves from 5-week-old Nicotiana benthamiana grown under 12h light/ 12h dark at 22°C were infiltrated with Agrobacterium expressing *pABindGFP-UBP12* or *pABindGFP-UBP12^{C208S}* with nuclear marker *pABindcherry-AS2* on separate plasmids. (a) Two representative image sets from each transfection combination show the localization of UBP12-GFP and UBP12^{C208S}-GFP along with the AS2-mCherry localization control. The scale bar indicates 50µm. (b) Schematic demonstrating quantitation strategy for nuclear GFP signal. Images were gathered using the same settings. Nuclear fluorescence was quantified by drawing two separate boxes in the nucleus, avoiding the periphery and nucleolus. The mean intensities of the two boxes were averaged to get an estimate of the nuclear GFP fluorescence (plotted in panel c). (c) Plot of the averages of the two mean intensities of the nuclear GFP signals from the control (no GFP), UBP12-GFP, and UBP12^{C208S}-GFP. Error bars represent standard deviation. No significant difference was found between the averages of the wild type and C208S mutant intensities using a two-tailed Welch's t-test (*p*-value=0.864604). The source data are provided as a Source Data file.

Supplementary Table 1. List of primers for cloning and qRT-PCR.

Primer Description	Sequences
**Cloning**	
pENTR-GI-Fw	CACCATGGCTAGTTCATCTTCATCTGAG
pENTR-GI-Rv	TTATTGGGACAAGGATATAGTACAGC
pENTR-GI-NS-Rv	TTGGGACAAGGATATAGTACAGC
pENTR-UBP12-Fw	CACCATGACTATGATGACTCCGCCTC
pENTR-UBP12-Rv	CTAATTGTATATTTTTACCGGCTTCT
pENTR-UBP12-NS-Rv	ATTGTATATTTTTACCGGCTTCT
pENTR-UBP13-Fw	CACCATGACTATGATGACTCCGCCGCCGCT
pENTR-UBP13-Rv	CTAATTGTATATTTTCACCGGCTTCTC
pENTR-UBP13-NS-Rv	ATTGTATATTTTCACCGGCTTCTC
UBP12-aa52-Rv	AGGAGGATCCTCAGCTGGCTGGTTC
UBP12-aa187-Rv	CCAGTAATCAAGAACCTTACGTACA
UBP12-aa530-Rv	GATTATTTTATCTTTGTCACTTTCTCGGA
UBP13-aa52-Rv	TGGAGGATCCTCGGGTGGTGGA
UBP13-aa187-Rv	CCAATAATCAAGAACTTTACGCACA
UBP13-aa529-Rv	GATTATCTTATCCTTGTCACTTTCCCGA
pENTR-UBP12-aa188-Fw	CACCTCATATGACTCTAAAAAAGAGACTGGTTTTGT
pENTR-UBP13-aa188-Fw	CACCTCATATGACTCAAAAAAAGAGACAGGTTTTGT
UBP12-C208S-SDM-Fw	CTCAAGAACCAAGGTGCAACAAGCTACATGAATTCTC
UBP12-C208S-SDM-Rv	GAGAATTCATGTAGCTTGTTGCACCTTGGTTCTTGAG
KpnI-UBP12-promoter-Fw	GGTACCACCCAATGATAAGTCTTTCTACCTACCCA
XhoI-UBP12-promoter-Rv	CTCGAGTGGCCGGAGAAGGATTAGACGGTGGGAT
**qRT-PCR**	
IPP2(AT3G02780)-Fw	ATTTGCCCATCGTCCTCTGT
IPP2(AT3G02780)-Rv	GAGAAAGCACGAAAATTCGGTAA
CCA1(AT2G46830)-Fw	TCTGTGTCTGACGAGGGTCGAATT
CCA1(AT2G46830)-Rv	ACTTTGCGGCAATACCTCTCTGG
ZTL(AT5G57360)-Fw	GTCAGAATGCATGGGGAAGT
ZTL(AT5G57360)-Rv	CGAGAAGGCTCAACAGAACC
HA-Fw	GGACTACGCTTCTTTGGGTGG
HA-Rv	GGATAGCCCGCATAGTCAGGAAC
YFP-Fw	CTTCAAGGACGACGGCAACTAC
YFP-Rv	TTCAGCTCGATGCGGTTCAC

Supplementary Table 2. Results of LS Periodogram analysis of the qRT-PCR data from figure 2 e-h and Supplementary Figure 3

Analysis of data from figure 2e and figure 2f

**Group Label**	**N**		**Period**	**Period Std**		**Abs Phase**	**Abs Phase Std**		**Amplitude**	**Amplitude Std**
Col-0	3		23.96	0.44		0.57	0.84		1.1	0.06
ubp12-1	3		21.87	0.2		21.42	0.48		1.22	0.07
gi-2	3		20.91	0.44		16.94	1.36		0.58	0.12
gi-2/ubp12-1	3		21.76	0		18.72	0.15		0.45	0.1
ubp13-1	3		23.29	0.28		23.01	1.23		1.23	0.08
gi-2/ubp13-1	3		22.66	0.44		22.15	1.06		0.56	0.13

Analysis of data from figure S3

**Group Label**	**N**		**Period**	**Period Std**		**Abs Phase**	**Abs Phase Std**		**Amplitude**	**Amplitude Std**
Col-0	3		24.2	0.21		0.13	0.65		1.43	0.17
ubp12-2w	3		20.61	0.62		17.66	2.01		1.06	0.02
gi-2	3		21.29	0.3		15.96	0.82		0.56	0.12
gi-2/ubp12-2w	3		20.72	0.62		17.27	1.64		0.45	0.11

Analysis of data from figure 2g and figure 2h

**Group Label**	**N**		**Period**	**Period Std**		**Abs Phase**	**Abs Phase Std**		**Amplitude**	**Amplitude Std**
Col-0	3		24.07	0.13		0.14	0.34		1.37	0.1
ubp12-1	3		22.29	0.31		-0.19	0.81		1.27	0.09
ztl-4	3		26.67	0.78		5.46	1.8		0.65	0.07
ztl-4/ubp12-1	3		24.81	0.15		3.78	0.43		0.72	0.07
ubp13-1	3		24.07	0.05		-0.69	0.07		1.32	0.02
ubp13-1/ztl-4	3		24.31	0.28		3.94	0.86		0.77	0.07
