# Supplementary material for: GIGANTEA recruits the UBP12 and UBP13 deubiquitylases to regulate accumulation of the ZTL photoreceptor complex
Source: Nat Commun. 2019 Aug 21;10:3750. doi: 10.1038/s41467-019-11769-7 (PMC6704089; doi:10.1038/s41467-019-11769-7)
Supplement: Supplementary file 3 — Description of Additional Supplementary Files [file 41467_2019_11769_MOESM3_ESM.pdf]

Description of Additional Supplementary Files

File Name: Supplementary Data 1

Description: The list of proteins identified by immunoprecipitation followed by mass spectrometry using 35S::FLAG-His-ZTL decoy in the Col-0 or gi-2 background.
