# Supplementary material for: GIGANTEA recruits the UBP12 and UBP13 deubiquitylases to regulate accumulation of the ZTL photoreceptor complex
Source: Nat Commun. 2019 Aug 21;10:3750. doi: 10.1038/s41467-019-11769-7 (PMC6704089; doi:10.1038/s41467-019-11769-7)
Supplement: Supplementary file 5 — Reporting Summary [file 41467_2019_11769_MOESM5_ESM.pdf]

nature research

Corresponding author(s): Joshua Gendron

Last updated by author(s): Jun 3, 2019

# Reporting Summary

Nature Research wishes to improve the reproducibility of the work that we publish. This form provides structure for consistency and transparency in reporting. For further information on Nature Research policies, see Authors & Referees and the Editorial Policy Checklist .

## Statistics

For all statistical analyses, confirm that the following items are present in the figure legend, table legend, main text, or Methods section.

| n/a | Confirmed | |
|---|---|---|
| ☐ | ☒ | The exact sample size (*n*) for each experimental group/condition, given as a discrete number and unit of measurement |
| ☐ | ☒ | A statement on whether measurements were taken from distinct samples or whether the same sample was measured repeatedly |
| ☐ | ☒ | The statistical test(s) used AND whether they are one- or two-sided<br>*Only common tests should be described solely by name; describe more complex techniques in the Methods section.* |
| ☒ | ☐ | A description of all covariates tested |
| ☒ | ☐ | A description of any assumptions or corrections, such as tests of normality and adjustment for multiple comparisons |
| ☐ | ☒ | A full description of the statistical parameters including central tendency (e.g. means) or other basic estimates (e.g. regression coefficient) AND variation (e.g. standard deviation) or associated estimates of uncertainty (e.g. confidence intervals) |
| ☒ | ☐ | For null hypothesis testing, the test statistic (e.g. *F*, *t*, *r*) with confidence intervals, effect sizes, degrees of freedom and *P* value noted<br>*Give P values as exact values whenever suitable.* |
| ☒ | ☐ | For Bayesian analysis, information on the choice of priors and Markov chain Monte Carlo settings |
| ☒ | ☐ | For hierarchical and complex designs, identification of the appropriate level for tests and full reporting of outcomes |
| ☒ | ☐ | Estimates of effect sizes (e.g. Cohen's *d*, Pearson's *r*), indicating how they were calculated |

*Our web collection on statistics for biologists contains articles on many of the points above.*

## Software and code

Policy information about availability of computer code

| | |
|---|---|
| Data collection | Image Lab, NIS-Elements, Micromanager, Bio-Rad CFX Manager, ZEN |
| Data analysis | FIJI, MASCOT, SAINTexpress on Spotlite, BRASS, Imager Lab |

For manuscripts utilizing custom algorithms or software that are central to the research but not yet described in published literature, software must be made available to editors/reviewers. We strongly encourage code deposition in a community repository (e.g. GitHub). See the Nature Research guidelines for submitting code & software for further information.

## Data

Policy information about availability of data

All manuscripts must include a data availability statement. This statement should provide the following information, where applicable:

- Accession codes, unique identifiers, or web links for publicly available datasets
- A list of figures that have associated raw data
- A description of any restrictions on data availability

The mass spectrometry proteomics data was deposited to the ProteomeXchange Consortium via the PRIDE partner repository (https://www.ebi.ac.uk/pride/archive/). It is accessible via identifier PXD014636.

The source data for figures 1, 2, and 3 and Supplementary Figures 3 and 4 are in the Source Data file. Additional data and materials reported in this study are available from the corresponding author upon request.

# Field-specific reporting

Please select the one below that is the best fit for your research. If you are not sure, read the appropriate sections before making your selection.

☒ Life sciences ☐ Behavioural & social sciences ☐ Ecological, evolutionary & environmental sciences

For a reference copy of the document with all sections, see nature.com/documents/nr-reporting-summary-flat.pdf

# Life sciences study design

All studies must disclose on these points even when the disclosure is negative.

| | |
|---|---|
| Sample size | The pre-determination of sample size was not calculated. The sample size of experiments reported in this study was based on common practice in this research filed. For the measurement of protein and mRNA levels with homogeneous population, n>8 were used for each sample. The circadian bioluminescent assays with the single ubp12 or ubp13 homogeneous mutant background, sample size 15 < n< 20 were used; whereas with the UBP12 T1 transgenic plants, larger sample size (n> 21) were chosen to determine the statistically significance of the noisy data. |
| Data exclusions | Data was not excluded from this study. |
| Replication | Experiments were performed with at least three biological biological repeats. Findings reported in this study were consistent among the replicates. |
| Randomization | Randomization was applied to circadian imaging experiments by varying location in the imager and varying location on plates of individual genotypes. |
| Blinding | Blinding was applied to all circadian imaging experiments. The person that collects and analyzes the data is given a code that is not representative of the material being analyzed. This ensures lack of bias in the analysis of the circadian imaging experiments. This is similar to the mass spectrometry experiments. |

# Reporting for specific materials, systems and methods

We require information from authors about some types of materials, experimental systems and methods used in many studies. Here, indicate whether each material, system or method listed is relevant to your study. If you are not sure if a list item applies to your research, read the appropriate section before selecting a response.

## Materials & experimental systems

| n/a | Involved in the study |
|---|---|
| ☐ | ☒ Antibodies |
| ☒ | ☐ Eukaryotic cell lines |
| ☒ | ☐ Palaeontology |
| ☐ | ☒ Animals and other organisms |
| ☒ | ☐ Human research participants |
| ☒ | ☐ Clinical data |

## Methods

| n/a | Involved in the study |
|---|---|
| ☒ | ☐ ChIP-seq |
| ☒ | ☐ Flow cytometry |
| ☒ | ☐ MRI-based neuroimaging |

## Antibodies

| | |
|---|---|
| Antibodies used | For immunoblotting, anti-HA-Biotin antibody (12158167001, Millipore-Sigma)<br>For immunoblotting, anti-ZTL antibody (from Dr. David Somers; Kim, W. Y., Geng, R. & Somers, D. E. Circadian phase-specific degradation of the F-box protein ZTL is mediated by the proteasome. Proc Natl Acad Sci U S A 100, 4933-4938, doi:10.1073/pnas.0736949100 (2003))<br>For immunoblotting, anti-GFP (ab-290, Abcam)<br>For immunoblotting,anti-FLAG antibody (F7425, Millipore-Sigma)<br>For immunoblotting,anti-Actin antibody (SAB4301137, Millipore-Sigma)<br>For Immunoblotting in co-IP experiments, anti-MYC antibody (C39656, Millipore-Sigma)<br>For Immunoblotting in co-IP experiments, anti-HA antibody (H3663, Millipore-Sigma)<br>For Immunoblotting in co-IP experiments, anti-FLAG antibody (F1804, Millipore-Sigma)<br>For Immunoblotting in co-IP experiments, anti-tubulin antibody (T5168, Millipore-Sigma) |
| Validation | These antibodies are either commercially available or kindly shared by Dr. David Somers. The validation of the antibodies were validated through the manufactures' websites or provided product data sheet. The validation of the anti-ZTL antibody can be found in the original paper listed above. |

## Animals and other organisms

Policy information about studies involving animals; ARRIVE guidelines recommended for reporting animal research

| | |
|---|---|
| Laboratory animals | n/a |
| Wild animals | n/a |
| Field-collected samples | This study involved model laboratory plants only. |
| Ethics oversight | Ethics oversight does not involve plants. |

Note that full information on the approval of the study protocol must also be provided in the manuscript.
